# MiR-711 and miR-183-3p as potential markers for vital reaction of burned skin

**DOI:** 10.1080/20961790.2020.1719454

**Published:** 2020-04-21

**Authors:** Kaikai Zhang, Ming Cheng, Jingtao Xu, Lijian Chen, Jiahao Li, Qiangguo Li, Xiaoli Xie, Qi Wang

**Affiliations:** a Department of Forensic Pathology, School of Forensic Medicine, Southern Medical University, Guangzhou, China; b Forensic Science Centre of Guangdong Provincial Public Security Department, Guangzhou, China; c Department of Critical Medicine, Mudan District People’s Hospital, Heze, China; d Department of Toxicology, School of Public Health, Southern Medical University, Guangzhou, China

**Keywords:** Forensic sciences, forensic pathology, vital reaction, skin burn, miR-711, miR-183-3p

## Abstract

In forensic practice, the identification of antemortem burns and postmortem burns is of the utmost importance. Reports from previous studies have shown that miRNAs, with lengths stretching over 18–25 nucleotides, are highly stable and resistant to degradation. However, there has been little research into the application of miRNAs in identifying antemortem and postmortem burns. This study compared the expression of miR-711 and miR-183-3p levels in mouse and postmortem human burned skins using RT-qPCR assay. RT-qPCR examination of burned mouse skins showed that increased miR-711 and miR-183-3p expression in comparison to intact skin tissues. The increased expressions of these two miRNAs were observed until 120 h after death in burned mouse skins, whereas no significant changes were found in postmortem burned skins. In human burned skins, the increased levels of these two miRNAs at 48 h following autopsy occurred in 19 of 26 subjects, which appeared to be related to the severity of the burn. These findings suggest that miR-711 and miR-183-3p may act as biomarkers for vital reaction of skin burn.

**Key points**
- This study investigated miR-711 and miR-183-3p levels in mouse and postmortem human burned skins using RT-qPCR.
- Increased miR-711 and miR-183-3p levels were observed in burned mouse skins.
- The increased expressions of these two miRNAs were observed until 120 h after death in burned mouse skin.
- The increased levels of these two miRNAs were observed until 48 h after autopsy in 19 of 26 forensic cases, which appeared to be related to the severity of the burn.

## Introduction

Wound examination is an important step, especially during the assessment of a burned body [1, 2]. Traditional methods of distinguishing antemortem burns from postmortem burns are based on the external and internal vital reactions, such as increased carbon monoxide-haemoglobin in circulation, erythema and blisters, soot deposits in respiratory and/or digestive lumen [3]. Those which are quickly workable have their drawbacks when it comes to some complex conditions: postmortem burns and putrefaction can also cause the appearance of erythema and blisters, and soot deposits and elevated carbon monoxide-haemoglobin can be absent in open areas [4]. In recent years, some studies have been conducted to determine wound vitality, though most of them mainly focused on proteins and mRNAs [5–10].

MicroRNAs (miRNAs/miRs), whose lengths are about 18–25 nucleotides, control gene expression by binding to the 3′-UTR of relevant mRNAs [11, 12]. Because of their wide distribution, easy storage, high conservation, and participation in various life activities, miRNAs have become a hot topic in clinical research [13]. However, there has been little research into the application of miRNAs in identifying antemortem and postmortem injuries.

The primary function of miR-711 is to modulate cell proliferation, apoptosis, as well as the cell cycle, and has become a probable biological target for the diagnosis and treatment of tumours [14, 15]. MiR-183 may be associated with the process of human non-small cell lung cancer and the regulation of the blood–brain barrier [16, 17]. Our previous study [1] used a miRNA microarray method to assess gene expression landscape of skin burn model. MiR-711 and miR-183-3p levels increased in antemortem burned skins. However, the application value of these miRNAs in forensic investigations has not been optimally exploited.

Therefore, the levels of miR-711 and miR-183-3p in human and mouse burned skins were explored and the utility of these miRNAs as vitality indicators of burned skins was evaluated.

## Materials and methods

### Animal models establishment

Male BALB/c mice (7–9 weeks old; (25 ± 3)g) were purchased from the Animal Centre of Southern Medical University (Guangzhou, China). A burned skin model was created as detailed in a prior study [1]. After the deep partial-thickness burn generated for 30 min, all mice were sacrificed with an overdose of anesthesia (60 mg/kg i.p.) and maintained at 24 °C. A 5 mm × 5 mm burned skin patch was cut at 0, 6, 12, 24, 48, 72, 96, and 120 h time-points (three mice per group). Skins obtained from unburnt mice served as the control (three mice per group). To obtain postmortem burn samples, after shaving the dorsal hairs, the mice were killed immediately (three mice). Dorsal skin burns were made 30 min after death with the same heated sheet of copper for 4 s. Then the postmortem burned skin specimen was harvested and stored in liquid nitrogen at −80 °C.

### Human autopsy specimen

Twenty-six human burned skin specimen harvested from forensic autopsy cases, with eight from females and 18 from males. The subjects’ age ranged from 23 to 55 years old. Intact regions without burning or other kinds of injuries were harvested from shoulder or abdominal skins of the same subjects as the control samples. Information concerning all subjects is presented in Supplementary material 1. First-degree burns are often superficial and appear reddish without blisters. Second-degree burns display erythema and skin blistering. For third-degree burns, substantial necrosis is observed in the epidermis and dermis [2]. The period from the injury to the time of death was considered as the survival period. We defined warm time as the period from death to cold storage and postmortem interval as the period from death time to autopsy. Tissue histopathology was carried out as per the routine procedures. Each tissue sample was subcategorized into sections weighing 0.1 g each and stored in sterilized and 2.0 mL microtubes (BIO-BIK) at 24 °C for 0, 24 and 48 h. Thereafter, they were immediately frozen and stored.

### Analysis of gene expression by RT-qPCR

The Agilent 2100 Bioanalyzer (Agilent Technologies, Palo Alto, CA, USA) was used to determine the RNA integrity number (RIN). Subsequently, cDNA was synthesized from the RNA using the PrimeScript RT reagent Kit (TaKaRa, Shiga, Japan). RT-qPCR was run on the Illumina Eco Real-Time PCR System (San Diego, CA, USA) under standard conditions. The mRNA level was calculated and normalized to the mRNA level of U6 using 2^−△△Ct^ method. All reactions, protocols, and conditions are provided in Supplementary materials 2 and 3.

### Statistical analysis

The gene expression products were assessed as triplicates and presented as mean ± SEM. Individual groups were compared using the non-parametric Mann–Whitney *U* test. Spearman’s rho method was employed for correlation analysis of paired parameters. All statistics were performed using GraphPad Prism version 5.01 (GrophPad Software, San Diego, CA, USA). *P*-value <0.05 was considered significant.

## Results

### Mice skin tissues

**Figure 1.**
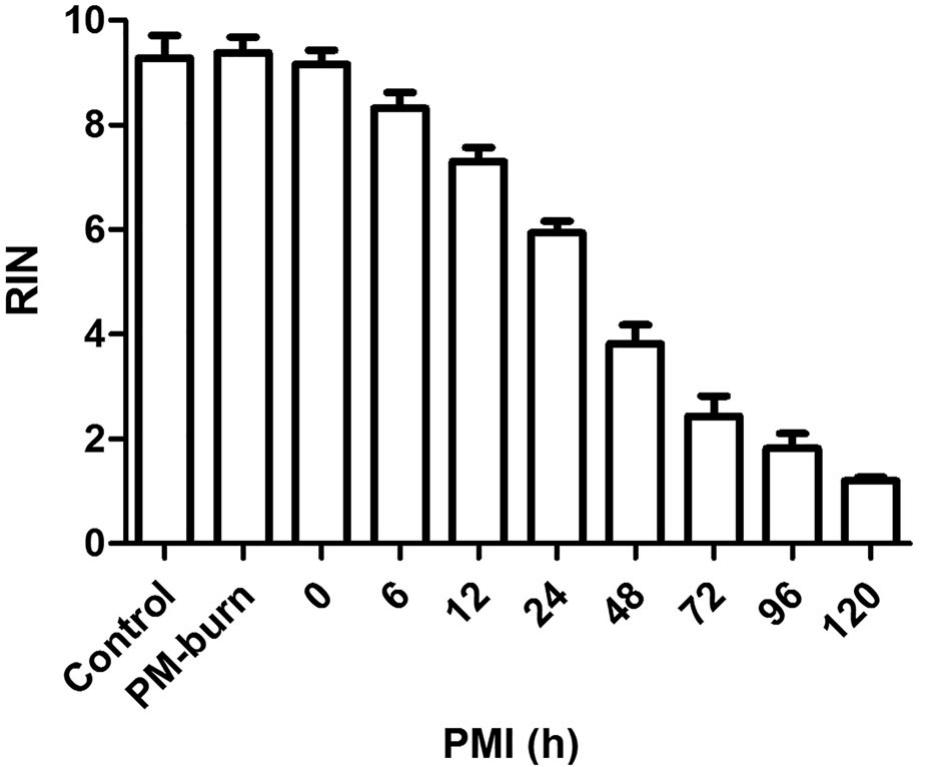
Postmortem interval-dependent changes of RNA integrity number (RIN) in mice skin specimen. RIN values showed no difference among the control, antemortem burn and postmortem burn groups, but showed a postmortem interval-dependent reduction in burned mice skin tissues. PM-burn: postmortem burn; PMI: postmortem interval.

Data analysis revealed that RIN values were not significantly different between postmortem burn, control, and antemortem burn groups. However, a postmortem interval-dependent decrease in RIN was observed in the burned mice skin specimen (Figure 1).

**Figure 2.**
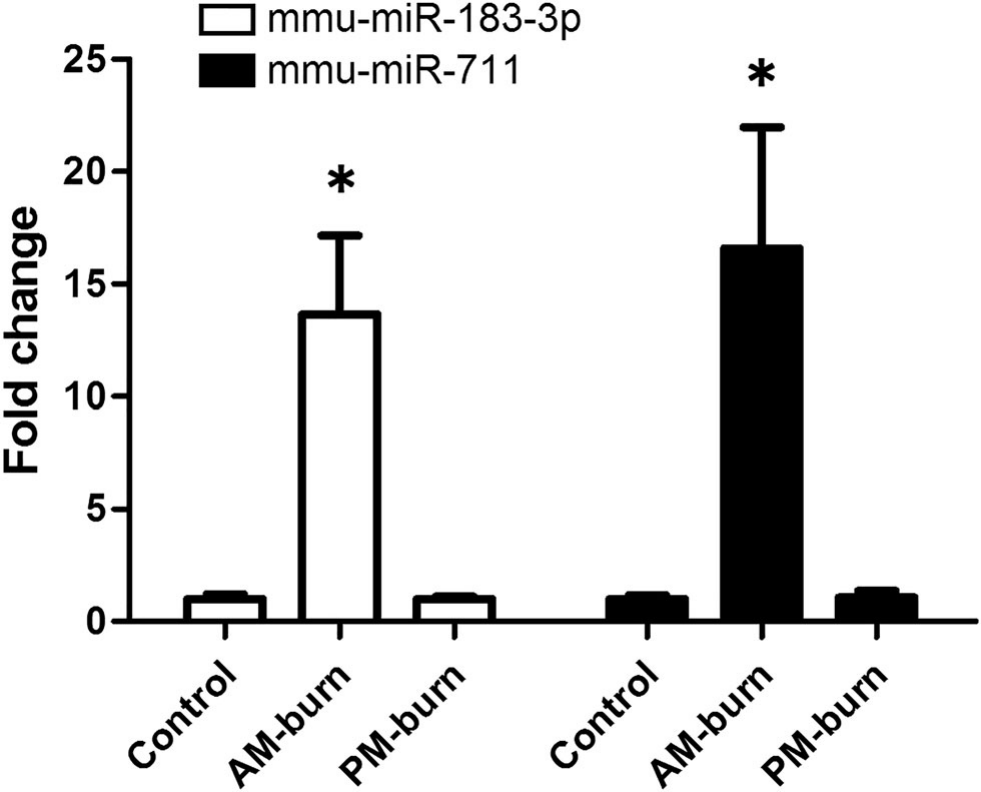
MiR-183-3p and miR-711 levels in mice skin tissues. *Significantly higher (*P* < 0.05), AM-burn *vs.* Control and PM-burn. AM-burn: antemortem burn; PM-burn: postmortem burn. mmu: *Mus musculus*.

Higher levels of miR-711 and miR-183-3p were recorded in burned skins in comparison with the control and postmortem burned regions (Figure 2).

**Figure 3.**
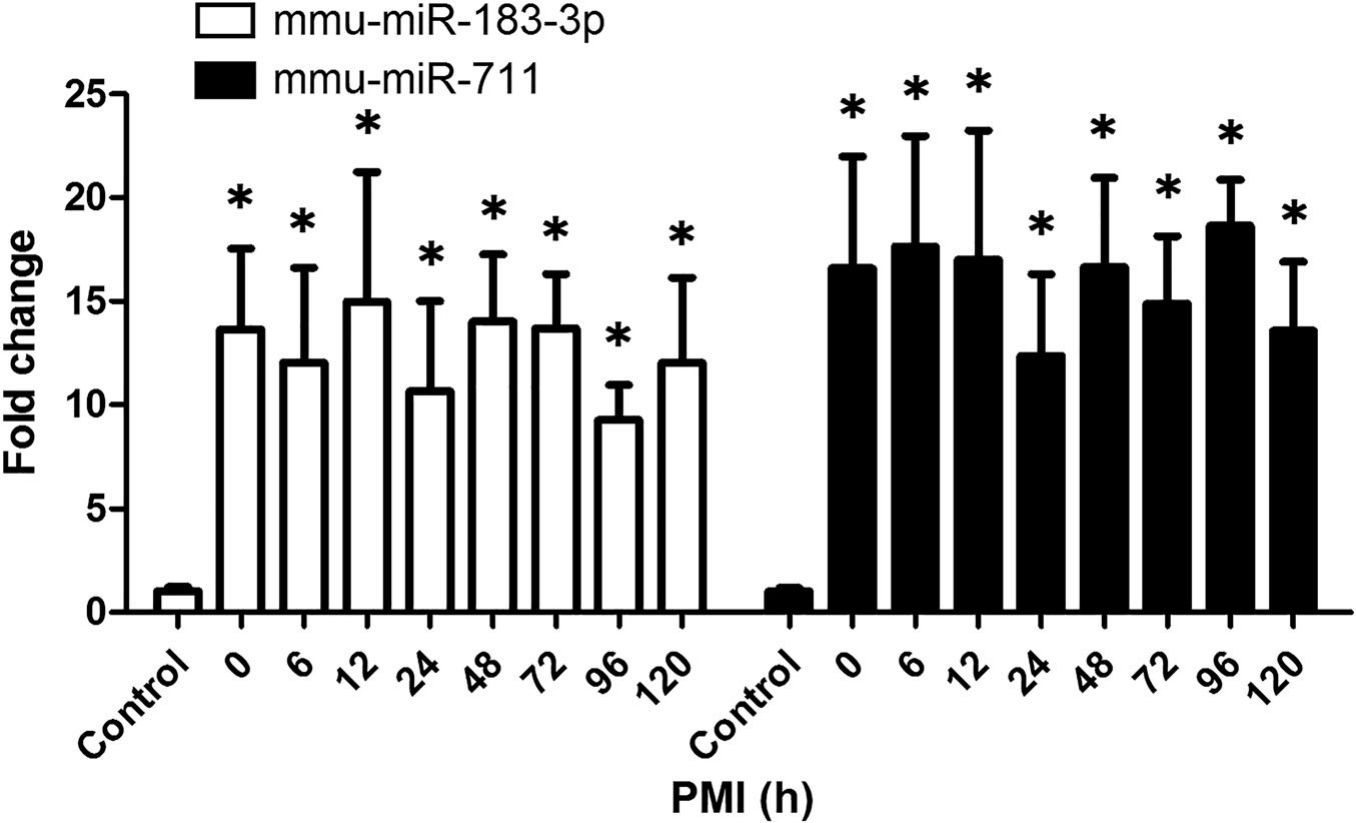
The impact of postmortem intervals on miR-183-3p and miR-711 levels in mice skin specimen. The elevated miR-183-3p and miR-711 levels in the burned skins were observed within 120 h after death. *Significantly higher (*P* < 0.05), compared with the control. mmu: *Mus musculus*.

We further examined whether postmortem intervals regulated miR-711 and miR-183-3p levels. Increased miR-711 and miR-183-3p levels in the burned regions were observed within 120 h after death (Figure 3).

### Postmortem human skin samples

Distinct differences were observed in RIN values among the groups but sex, age, postmortem interval, or warm time were not markedly different among the groups as evidenced by results of Spearman’s rho (*P* > 0.05).

**Figure 4.**
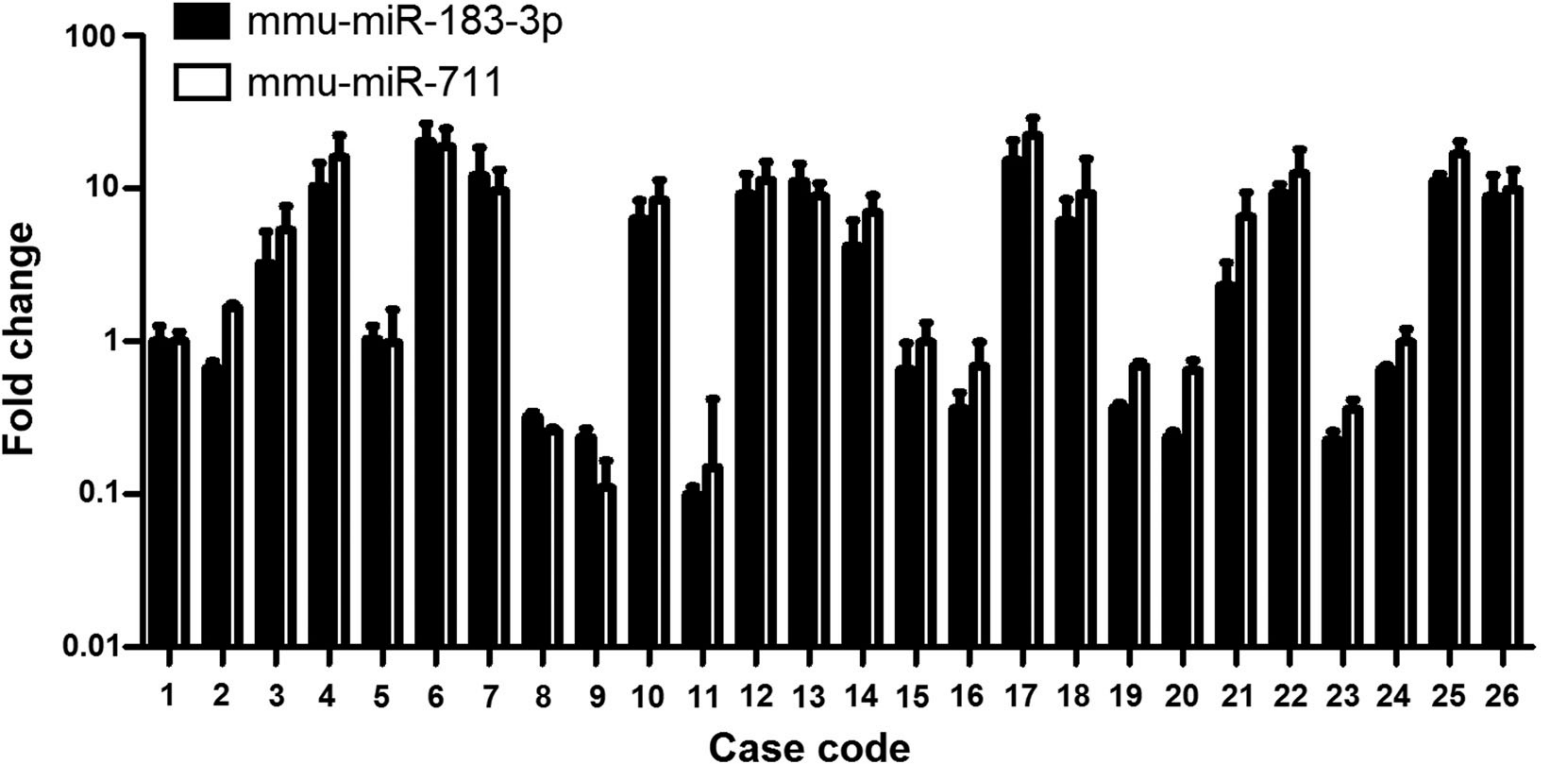
MiR-183-3p and miR-711 levels showed relatively high inter-individual differences in intact regions. mmu: *Mus musculus*.

MiR-711 and miR-183-3p levels revealed considerable inter-individual variations in intact skin samples (Figure 4). Thus, fold changes of miR-711 and miR-183-3p levels in burned regions were compared with the intact skins from the same individuals.

**Figure 5.**
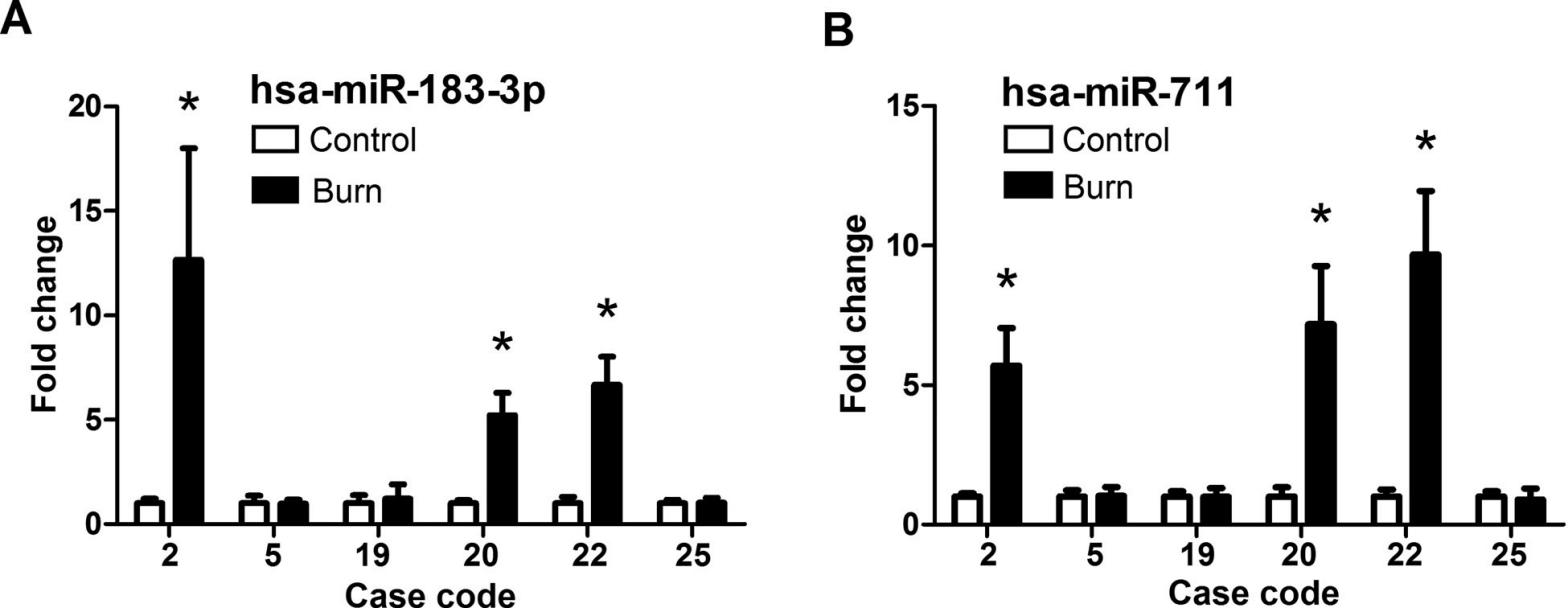
MiR-183-3p (A) and miR-711 (B) levels in postmortem human skin tissues with first-degree burn. *Significantly higher (*P* < 0.05), Burn *vs.* Control. hsa: *Homo sapiens*.

**Figure 6.**
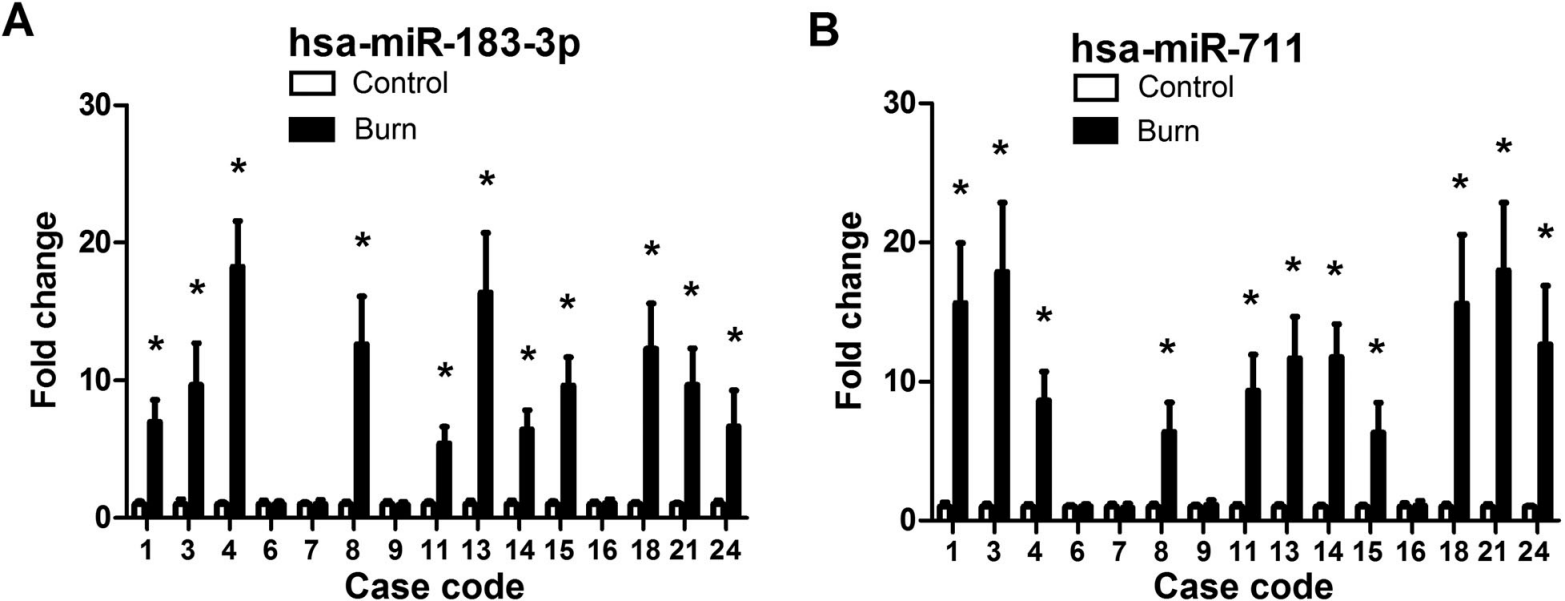
MiR-183-3p (A) and miR-711 (B) levels in postmortem human skin tissues with second-degree burn. *Significantly higher (*P* < 0.05), Burn *vs.* Control. hsa: *Homo sapiens*.

**Figure 7.**
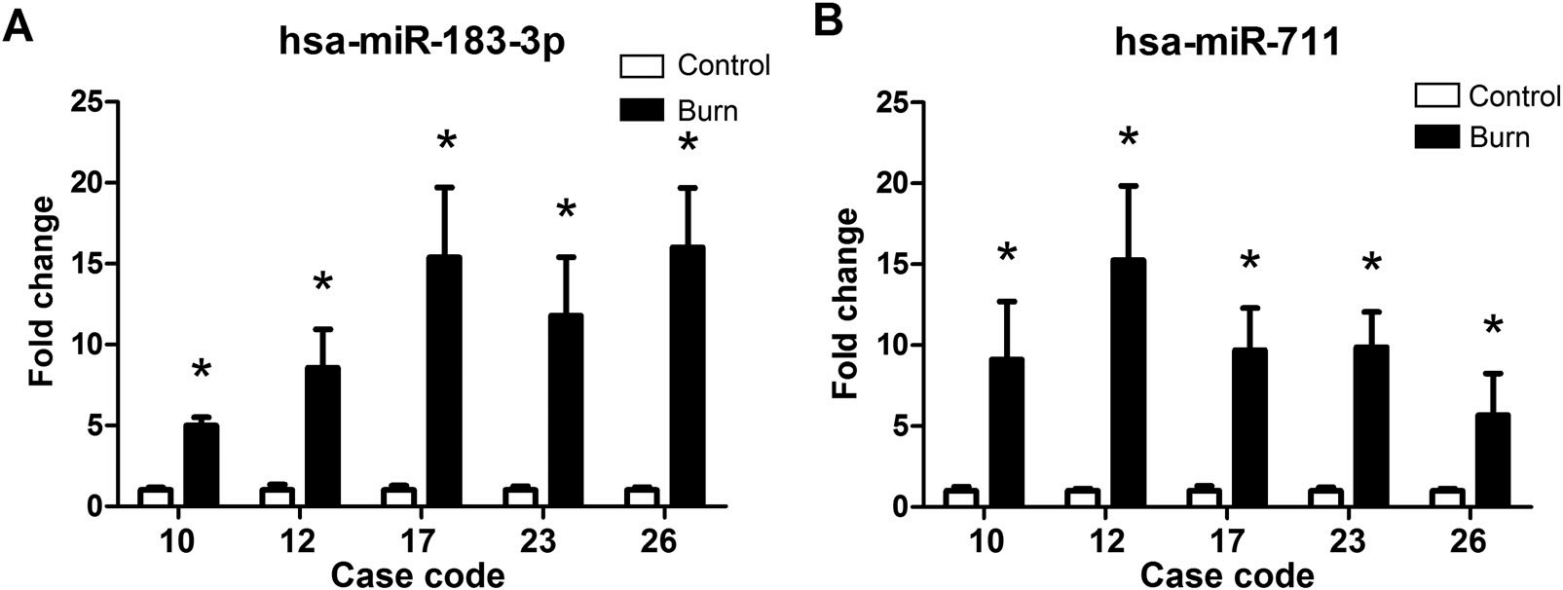
MiR-183-3p and miR-711 levels in postmortem human skin tissues with third-degree burn. *Significantly higher (*P* < 0.05), Burn *vs.* Control. hsa: *Homo sapiens*.

In burned regions, increased miR-711 and miR-183-3p levels were observed in 19 of 26 cases. For first-degree burn samples, miR-711 and miR-183-3p levels were increased in three of six cases (Figure 5). For second-degree burn samples, miR-711 and miR-183-3p levels were increased in 11 of 15 cases (Figure 6). For third-degree burn samples, these miRNAs were up-regulated in all five cases (Figure 7).

**Figure 8.**
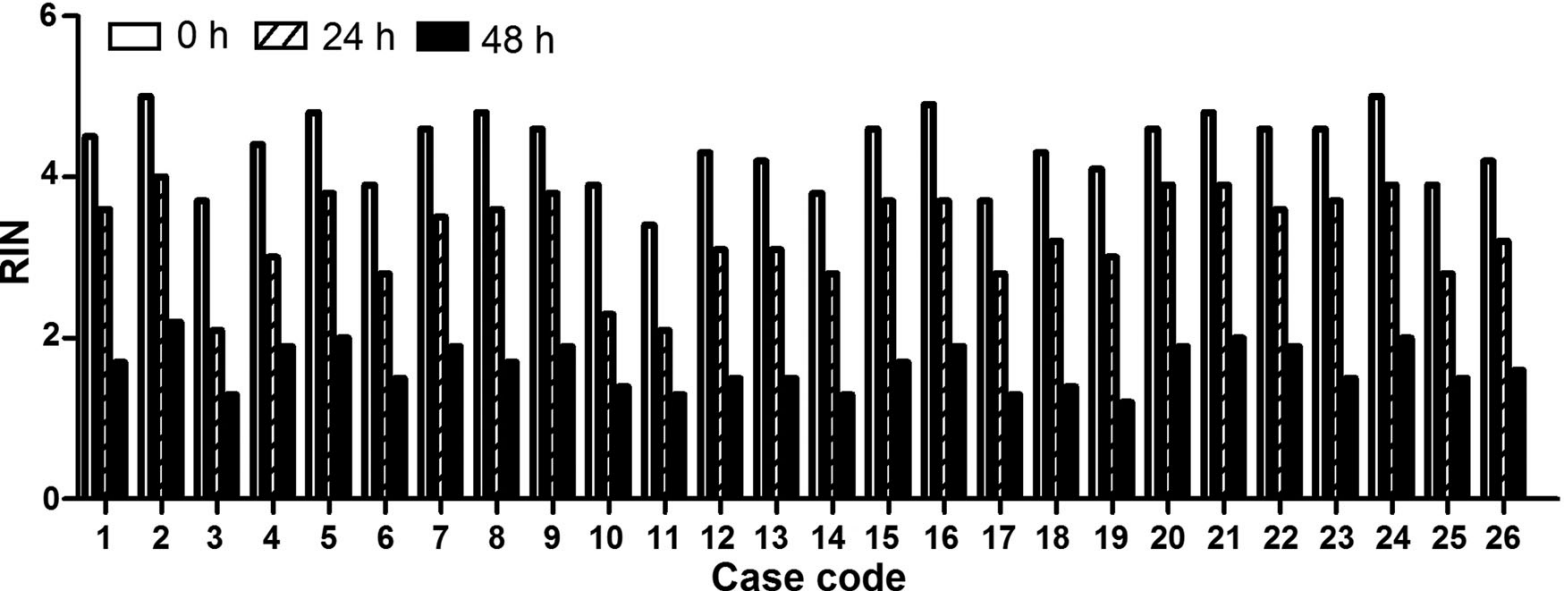
Postmortem interval-dependent changes of RNA integrity number (RIN) in postmortem human skin tissues. RIN values showed a postmortem interval-dependent reduction in burned human skin tissues.

The effects of postmortem intervals were also evaluated. RIN values showed a postmortem interval-dependent reduction in burned human skin tissues (Figure 8). Increased miR-711 and miR-183-3p levels were detected until 48 h after autopsy in all the above-mentioned 19 cases (Supplementary materials 4–6).

## Discussion

The identification of antemortem and postmortem burns is extremely important for the field of forensic pathology. When a corpse is found at a fire scene, a forensic pathologist needs to find out whether the person was alive upon sustaining the burns and determine whether the death occurred due to thermal injury. However, conventional findings to identify antemortem burns are usually unspecific, or even absent. Therefore, novel markers for burn vitality are needed. Previous studies, using the immunohistochemical method, reported that increased P-selectin, fibronectin, heat-shock protein 70, von Willebrand factor, and PECAM-1 were detected in the respiratory tract and lungs of fire victims, which support intravital reaction in fatal burns [5, 10, 18, 19]. Kubo et al. [4] reported that the increased mRNA expression of AQP3 was observed in antemortem burn skin.

MiRNAs are very short nucleic acids which are resistant to extreme temperatures and pH. Some studies have reported that miRNAs are stable at room temperature and can be identified after long-term fixation [20]. Some miRNAs even remain detectable in storage at −20 °C for 10 years [21, 22].

In our previous study [1], an miRNA microarray technique was used to determine the miRNA expression profiles. A total of 24 differentially expressed miRNAs were observed in burned mice skins compared to that in unburned skins. Among these differentially expressed miRNAs, levels of miR-711 and miR-183-3p were significantly increased. This study reproduced the burned skin model, and evaluated the application of miR-711 and miR-183-3p as potential markers for vital reaction.

In our animal experiment, we found that miR-711 and miR-183-3p levels were higher in the antemortem burned regions than those in the intact skin samples. In addition, postmortem burn did not induce changes in miR-711 and miR-183-3p levels in mouse skins, suggesting that these two miRNAs are potential biomarkers for differentiating antemortem from postmortem burns.

In contrast to mice specimen, tissues obtained during autopsy are usually affected by postmortem intervals [23]. The effects of putrefaction and autolysis should, therefore, not be ignored. Therefore, the effects of postmortem intervals on these two miRNAs were also investigated. RIN is an indicator for assigning total RNA integrity [24]. In this study, RIN values tended to decrease in a postmortem interval-dependent manner in mice skin specimen, showing considerable degradation of RNA. However, the increased miR-711 and miR-183-3p levels can still be observed until 120 h after death. These results showed that RNA integrity was compromised by postmortem intervals, but miR-711 and miR-183-3p were considerably stable, at least for 120 h after death.

Twenty-six human skin tissues were used to validate the mice model results. These two miRNAs levels were markedly higher in burned regions of 19 cases relative to intact regions. These significant changes can still be observed until 48 h after autopsy. Interestingly, the increased miRNAs seemed to be related to burn degree. The increased miR-711 and miR-183-3p levels were observed in three of six cases for first-degree burn samples, in 11 of 15 cases for second-degree burn samples, and in all five cases for third-degree burn samples. Further studies should therefore explore the underlying mechanisms.

MiR-711 has been shown to be mainly involved in cell proliferation, apoptosis and regulation of the cell cycle. Its up-regulation suggests that it may participate in the repair of skin tissue after burns and promote the apoptosis of injured cells. MiR-183-3p has been reported to be involved in the opening of the blood–brain barrier. In this study, it may be related to the regulation of skin capillary permeability after burns and increase the biological functions, such as inflammatory factor exudation. These may be the possible reasons for the significant increase of these two miRNAs after burns, which still require further investigation.

In conclusion, the present study using mouse and postmortem human burned skin tissues suggests that the detection of miR-711 and miR-183-3p levels might be useful for the determination of vital reaction of burned skin.

## Authors’ contributions

Kaikai Zhang and Ming Cheng carried out the genetic studies and participated in the miRNA microarray analysis; Jingtao Xu and Jiahao Li carried out the RT-qPCR and performed the statistical analysis; Lijian Chen and Qiangguo Li established the animal model and drafted the manuscript; Xiaoli Xie conceived of the study and participated in its design; Qi Wang participated in coordination and helped to draft the manuscript. All authors contributed to the final text and approved it.
